# Low temperature-induced variation in plasma biochemical indices and aquaglyceroporin gene expression in the large yellow croaker *Larimichthys crocea*

**DOI:** 10.1038/s41598-018-37274-3

**Published:** 2019-02-25

**Authors:** Cheng Liu, Weiliang Shen, Congcong Hou, Xinming Gao, Qianfeng Wang, Xiongfei Wu, Junquan Zhu

**Affiliations:** 1 Key Laboratory of Applied Marine Biotechnology of Ministry of Education, School of Marine Sciences, Ningbo University, Ningbo, Zhejiang, 315211 China; 2 Ningbo Academy of Oceanology and Fishery, Zhejiang, 315012 China

## Abstract

Low temperature influences multiple physiological processes in fish. To explore the adaptability of the large yellow croaker (*Larimichthys crocea*) to low temperature, the concentrations of glycerol, blood urea nitrogen (BUN), and triglycerides (TG) in plasma, as well as the expression levels of metabolism-related genes *aqp7* and *aqp10*, were measured after exposure to low temperature stress and during subsequent rewarming. In addition, tissue samples from the intestine and liver were histologically analyzed. We found that the concentrations of plasma glycerol, BUN, and TG, decreased under low temperature stress, suggesting the metabolism of fat and protein slowed at low temperature. The expression levels of *aqp7* and *aqp10* mRNA were also downregulated under exposure to low temperature. Interestingly, above plasma indices and gene expression returned to basic levels within 24 h after rewarming. Furthermore, the liver and the intestine were damaged under continuous low temperature stress, whereas they were repaired upon rewarming. From the above results, we concluded that *aqp7* and *aqp10* genes were sensitive to low temperature, and that the decrease in their expression levels at low temperature might reduce energy consumption by *L*. *crocea*. However, the adaptation to low temperature was limited because the key metabolic tissues were damaged under continuous exposure to low temperature. Interestingly, the metabolism of *L*. *crocea* was basically back to normal within 24 h of rewarming, showing that it has high capacity of self-recovery.

## Introduction

Warm-temperature fish are generally in a negative energy balance due to the lack of natural food sources and decreased feeding ability in winter. In this case, triglycerides (TGs) in fish are hydrolyzed to form free fatty acids (FFA) and glycerol through adipose triglyceride lipase (ATGL) and hormone sensitive lipase (HSL), which are released into the blood to provide energy^1,2^. Many studies on fish such as *Cyprinus carpio*^3^, *Pseudosciaena crocea*^4^ and *Oreochromis niloticus*^5^ have reported that the concentration of TG in the blood increases at low temperature stress, indicating fat is mobilized and decomposed at low temperature, thus providing enough energy for fish to cope with low temperature. As an important product of fat metabolism, glycerol is not only used as substrate for gluconeogenesis in the liver, but it is also used as an energy substrate to produce ATP and provide energy through oxidative phosphorylation in mitochondria^2^. Therefore, the change of glycerol content reflects the change of lipid metabolism to some extent.

Carbohydrate intake in most fish is limited at low temperatures, in which case, amino acids become the main materials for glycerol synthesis^6^. Meanwhile, ammonia derived from amino acids is converted into urea in the process of glycerol synthesis, leading to an increase in blood urea content, which is beneficial for the removal of toxins from the body, playing a certain detoxification role and improving the viability of cold-water fish at low temperature^7,8^. Furthermore, as proteins are metabolized, the change in urea concentration in the plasma reflects the rate of protein metabolism to some extent. However, few studies have discussed the effect of low temperature on glycerol and urea metabolism in warm-water fish. As important organs for metabolism, liver and intestine are the main places of fat and protein metabolism. A clear understanding of the status of metabolism in fish requires a thorough study of the histomorphological changes of the liver and intestine that occur under low temperature.

The primary mechanism whereby glycerol and urea cross the plasma membrane is through transmembrane proteins belonging to the aquaporin family^9^. Aquaporins (AQPs) are constitutive membrane proteins that allow the movement of water and other small molecules (e.g., urea and glycerol) across the cell membrane, playing an important role in maintaining the homeostasis of the internal and external environment in cells^2,10,11^. There are 13 AQPs (AQP0 to AQP12) that mediate critical biological functions in mammals^12^. According to their structure and functions, AQPs can be classified into three subgroups (aquaporins, aquaglyceroporins, and superaquaporins). Among these, AQP3, 7, 9, and 10, called aquaglyceroporins, can transport some small molecules (e.g., glycerol, urea, and nitric oxide) across cell membranes, as well as facilitate the movement of water^10,13^. It has been reported that aquaglyceroporins can regulate the absorption and release of water and glycerol in tissues, thereby influencing energy metabolism^7,14^. Up to present, there have been few studies on the effect of low temperature on AQPs in fish (e.g., *Oreochromis niloticus*^15^, *Danio rerio*^16^, and *Osmerus mordax*^7^). Among AQPs, AQP7 and AQP10 are important channel proteins in the liver and intestines, respectively, that play an important role in regulating glycerol and urea metabolism in the organs in which they are found^17–19^. Therefore, to a certain extent, changes in expression level of *aqp7* and *aqp10* at low temperature reflect metabolic changes of fat and protein, which is a valuable reference for the study of energy metabolism in fish at low temperature.

The large yellow croaker (*Larimichthys crocea)*, is one of the most economically important marine, cultured teleost fish species in China^20^. The optimum growth temperature for *L*. *crocea* is 18–25 °C, whereas its energy metabolism is adversely affected at temperatures below 13 °C. Generally, in their natural environment *L*. *crocea* enter the deep sea to overwinter, while in coastal cage culture they are unable to go to the deep sea; consequently, large numbers die of cold death when an extreme low temperature event occurs^21^; furthermore, their own energy is mainly consumed to meet metabolic needs due to the lack of natural food sources and the decline in feeding ability in winter. In this case, metabolism of *L*. *crocea* must be adjusted to survive in low temperature environments for several months during winter. It has been found in our previous study that 9 °C is its semi-lethal temperature at 48 h of acute low temperature stress. Therefore, we explored the effect of acute low temperature (9 °C) stress and rewarming on the blood biochemical indices, histomorphology of the intestine and liver, and on the expression of aquaglyceroporin genes associated with energy metabolism. We believe our study will provide a sound theoretical basis for studying the adaptability of *L*. *crocea* to low temperature.

## Results

### Effects of acute low temperature stress and rewarming on plasma BUN, glycerol, and TG in *L*. *crocea*

**Figure 1:**
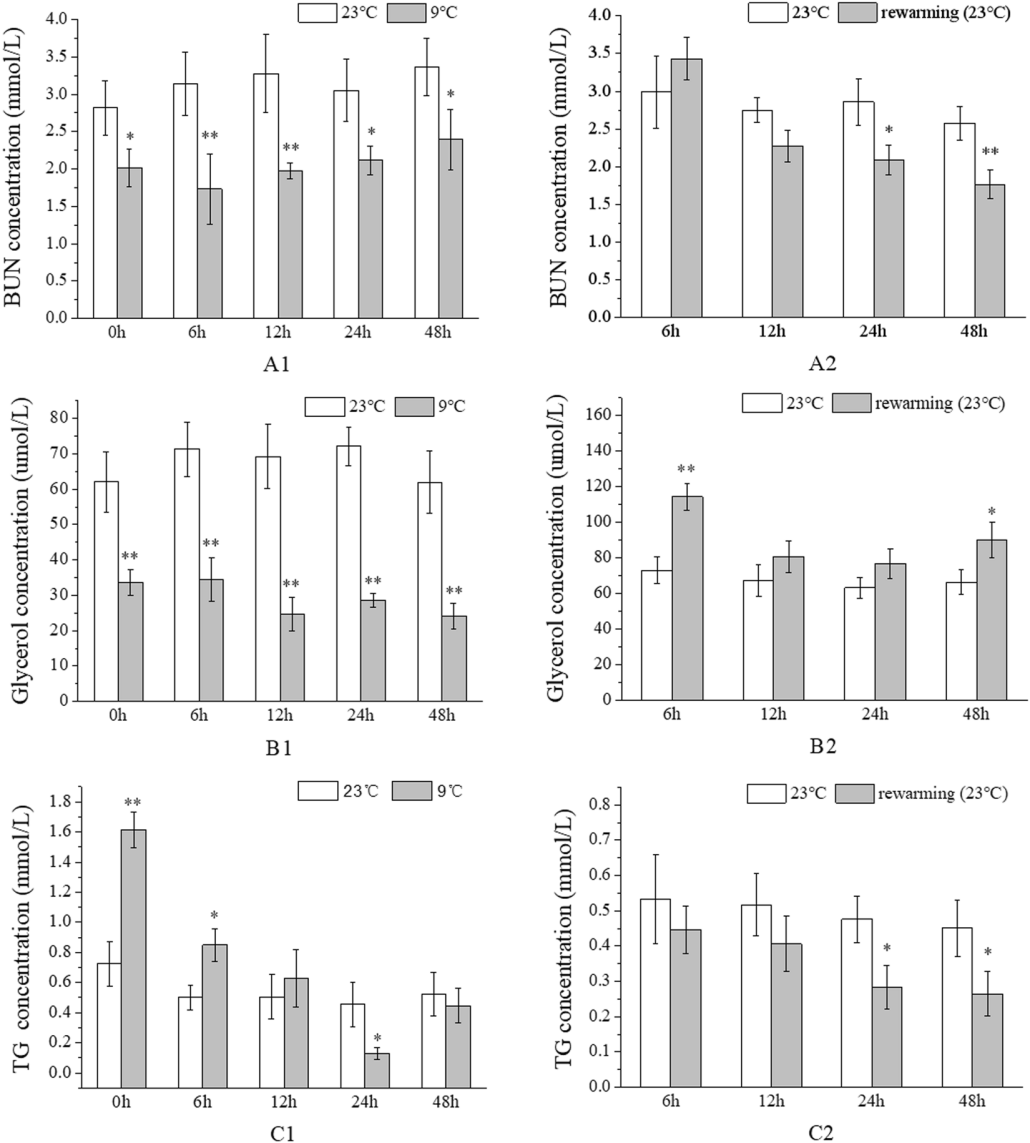
Effects of acute temperature changes on the concentration of plasma BUN, glycerol, and TG in *L*. *crocea*. (**A1**,**B1**,**C1**) The concentrations of BUN, glycerol and TG in the plasma varied with the time of low temperature stress, respectively. (**A2**,**B2**,**C2**) Concentrations of BUN, glycerol and TG in the plasma varied with the rewarming time, respectively. Date were shown as the mean ± SD (N = 3). Each experiment was performed in triplicate. Significant difference is indicated by an asterisk (*p* < 0.05) or a double asterisk (*p* < 0.01).

As shown in Fig. 1A1,B1, the concentrations of blood urea nitrogen (BUN) and glycerol in the plasma of fish under low temperature stress were significantly lower than those in the control group (*P* < 0.05); further, BUN concentrations first decreased and then increased, whereas the concentrations of plasma glycerol decreased gradually. In addition, the concentration of plasma TG in the low temperature group also decreased initially but then increased and was significantly higher than that in the control group at 0 h under low temperature stress; next it decreased to its lowest value at 24 h after treatment initiation, at which point it was significantly lower than the corresponding content in controls (*P* < 0.05) (Fig. 1C1).

During the rewarming period, the concentrations of plasma BUN and TG in the rewarming group decreased gradually, and basically returned to the levels of those in the control group at 12 h (*P* > 0.05), but they decreased significantly after 24 h (*P* < 0.05) (Fig. 1A2,C2). The concentration of plasma glycerol first decreased and then increased, and returned to the basal concentration in 12 h but was significantly higher than in the control group at 48 h after rewarming (*P* < 0.05) (Fig. 1B2).

### Expression of *aqp7* and *aqp10* mRNA in different tissues in the control group

**Figure 2:**
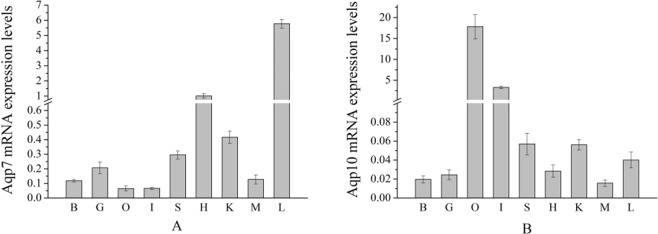
Expression profiles of *aqp7* (**A**) and *aqp10* (**B**) mRNA in control tissues, as assessed by qPCR. Expression levels were normalized against β-action in each sample. Abbreviations for tissues in x-axis are as follows: B: brain, G: gill, O: ovary, I: intestine, S: spleen, H: heart, K: kidney, M: muscle, L: liver.

The expression levels of *aqp7* and *aqp10* mRNA were determined for multiple tissues examined, including the brain, gill, heart, kidney, liver, spleen, intestine, gonads, and muscle, using a qPCR assay. The expression level of *aqp7* was highest in the liver, followed by the heart; while the lowest expression was detected in the ovary (Fig. 2A). In contrast, mRNA expression level of *aqp10* was relatively higher in the ovary and the intestine (Fig. 2B).

### Expression changes in *aqp7* and *aqp10* mRNA under acute temperature stress

**Figure 3:**
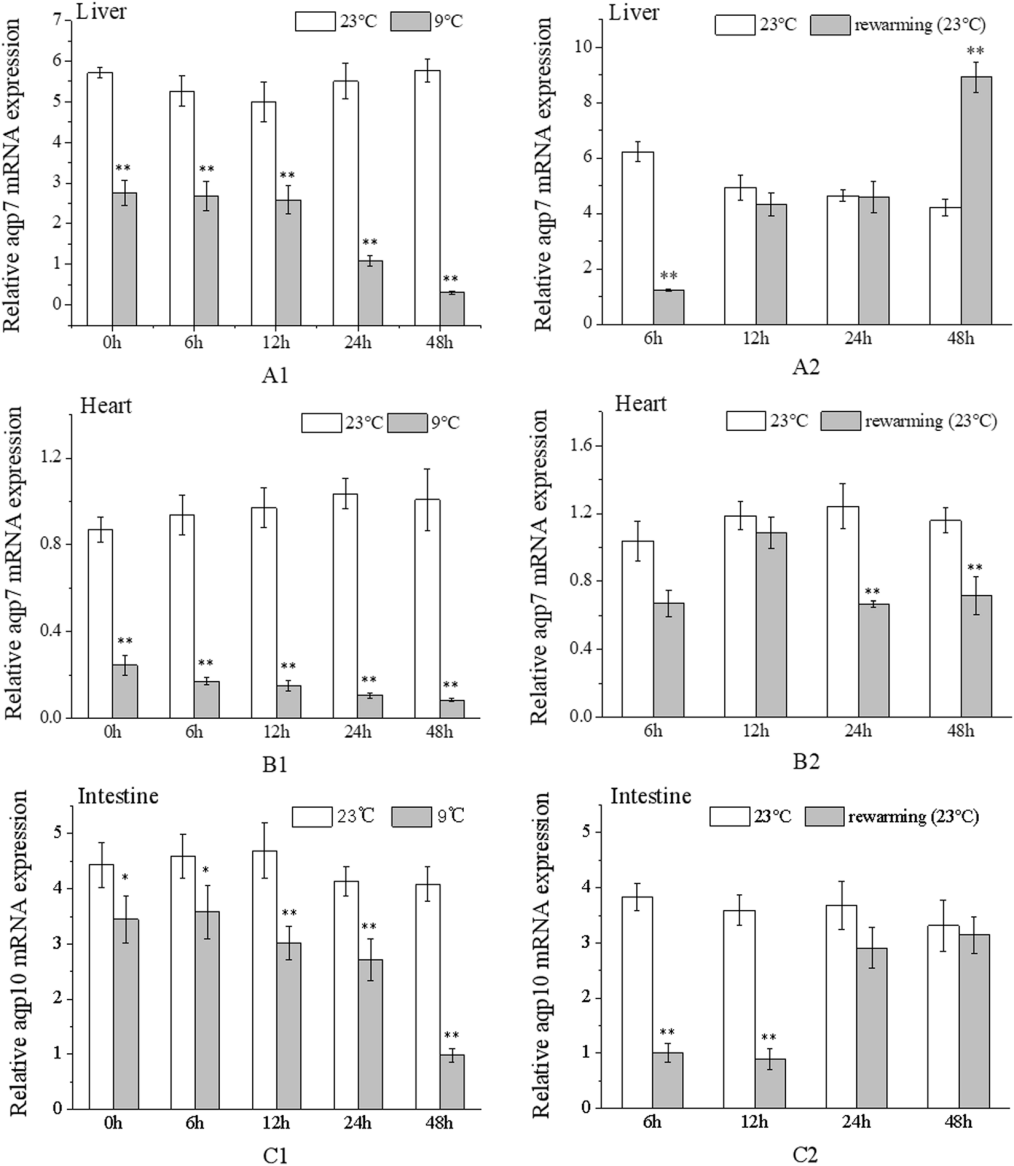
Expression levels of *aqp7* and *aqp10* mRNA in *L*. *crocea* under low temperature and subsequent rewarming. (**A1**,**B1**,**C1**) Expression levels of *aqp7* gene in the liver and heart, as well as *aqp10* in the intestine changed with time under low temperature stress. (**A2**,**B2**,**C2**) *aqp7* and *aqp10* mRNA expression levels varied with time of rewarming treatment.

Under low temperature stress, the expression levels of *aqp7* in both, liver and heart, as well as that of *aqp10* in the intestine, were mainly downregulated and significantly lower than those in the control group (*p* < 0.05) (Fig. 3A1,B1,C1).

During the rewarming period, *aqp7* mRNA expression level in the liver was upregulated, and was returned to the basal level at 12 h, but increased significantly after 48 h (*P* < 0.05) (Fig. 3A2). Conversely, the expression level of *aqp7* in the heart, first increased and then decreased and was returned to the control level at 6 h, whereas it was significantly lower than that of the control group after 24 h (*P* < 0.05) (Fig. 3B2). Similar to the expression level of *aqp7* in the liver, *aqp10* expression level gradually increased and was significantly lower than that in the control group at 6 and 12 h (*P* < 0.05), whereas, there was no significant difference in *aqp10* expression level between rewarming and control groups after 24 h (*P* > 0.05) (Fig. 3C2).

### Effects of acute temperature changes on the intestine and liver of *L*. *crocea*

**Figure 4:**
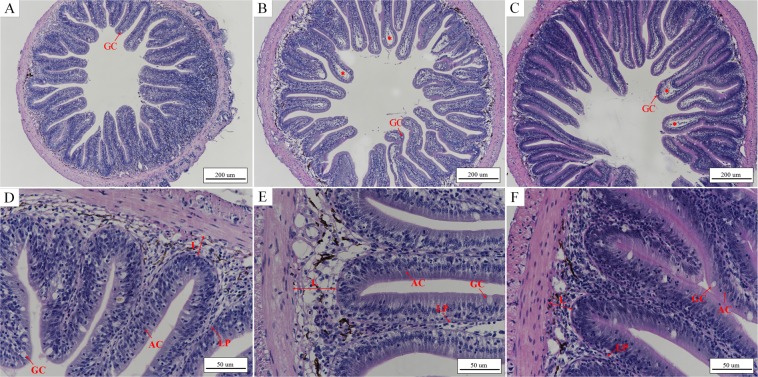
Morphological changes of the intestine of *L*. *crocea* under low temperature stress and during subsequent rewarming. (**A**,**D**) Intestine of the control group (×100 and ×400, respectively). The folds of intestinal mucosal were complete, and GCs were scattered among ACs. The submucosa was compact, and the lamina propria was not vacuolated. (**B**,**E**) Intestine of the low temperature group (×100 and ×400, respectively). The intestinal mucosa appeared partially broken, the submucosa became wider and the lamina propria became vacuolated, resulting in the increase of intestinal permeability. (**C**,**F**) Intestine of the rewarming group (×100 and ×400, respectively). The intestinal mucosa is still broken, vacuolization of the lamina propria is relatively reduced, and the number of GCs in local intestinal mucosa increased, compared with the low temperature (Table 1). Figure note: absorptive cell (AC), goblet cell (GC), lamina propria (LP), width of submucosa (L), vacuolization of lamina propria (asterisk).

**Figure 5:**
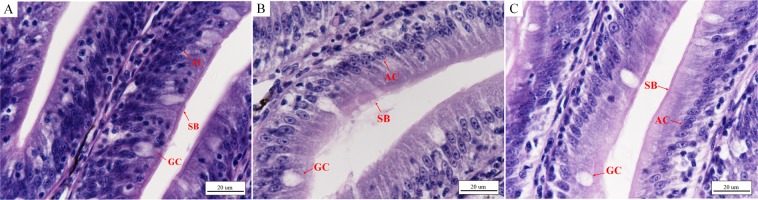
Detail changes of the intestines in *L*. *crocea* under low temperature and during subsequent rewarming. (**A**–**C**) Intestines of individuals in the control, low temperature, and rewarming groups (×1000), respectively. The free surface of ACs in the small intestinal epithelium has a clear striped border composed of microvilli called the SB, which acts as a mechanical barrier (**A**). At low temperature, part of the microvilli fell off and the SB became blurred (**B**), resulting in increased permeability of the intestine. During the rewarming period, the SB was still partially shedding (**C**). Figure note: striated border (SB), the others were shown in Fig. 4.

**Figure 6:**
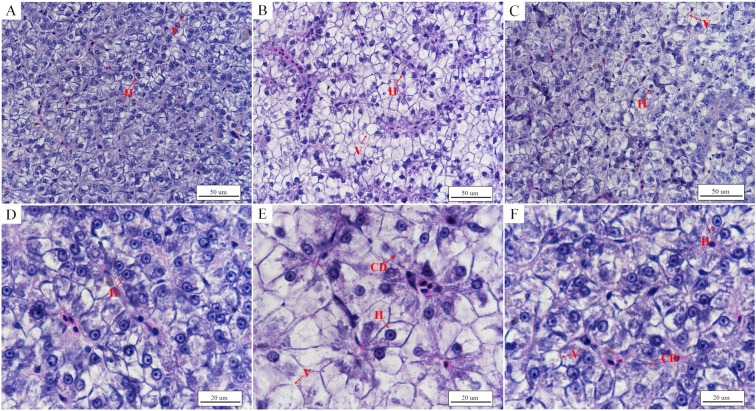
Morphological changes of liver of *L*. *crocea* under low temperature and during subsequent rewarming with HE staining. (**A**,**D**) Liver of individuals in the control group (×400 and ×1000, respectively). The hepatocyte density in the liver were relatively high and the nucleus was located on the side of the cell, with less vacuolization. (**B**,**E**) Liver of individuals in the low temperature group (×400 and ×1000, respectively). The density of hepatocytes decreased obviously, as well as cell swelling and vacuolization increasing, with cell membrane disruption of the local liver. (**C**,**F**) Liver of of individuals in the rewarming group (×400 and ×1000, respectively). The density of hepatocytes increased and vacuolization decreased, compared with the low temperature group. Figure note: Hepatocyte (H), vacuolation (V), cell disruption (CD).

There was a clear difference between fish with normal intestinal and liver structures in the control group and those with damaged intestines and livers after 48 h under low temperature stress. The number of goblet cells in the intestinal mucosa decreased after 48 h under low temperature stress (Table 1), with vacuolization of the lamina propria and breaking of folds in the mucous membrane (Fig. 4B). In addition, the submucosa was obviously widened, and the striated border of the mucous membrane was incomplete and partially shed (Figs 4E and 5B). Conversely, the intestinal mucosa in the control group was complete, and there was no vacuolization of the lamina propria (Figs 4A,D and 5A). The hepatocyte density in the low temperature group decreased, and partial hepatocytes swelled, with broken cells and vacuolization (Fig. 6B,E). In contrast, the hepatocyte density in the control group was relatively high, with a few examples of cell vacuolization (Fig. 6A,D).

**Table 1:** Number of goblet cells in the intestine of *L*. *crocea* (number of cells/mm^2^).

	control group	low temperature group	rewarming group
1	164	116	135
2	178	142	166
3	197	135	143
4	175	96	131
5	184	121	122
6	153	103	119
Mean ± SD	175 ± 15	119 ± 18^**^	136 ± 17^**^

At 48 h after rewarming initiation, the number of goblet cells (GCs) in the intestinal mucosa had increased (Table 1), and the striated border was still partially shed, with vacuolization of the lamina propria and narrowing of partial submucosa (Figs 4C,F and 5C), compared to those of the low temperature group (Figs 4E and 5B). The hepatocyte density increased, and the volumes of the cells decreased, with a relative decrease in vacuolated and broken cells (Fig. 6C,F), compared to those in the low temperature group (Fig. 6B,E).

## Discussion

Fish metabolism is strongly influenced by temperature fluctuations. It has been reported that fat is first decomposed to produce energy at low temperatures, maintaining the requirements for normal metabolism, but protein is mainly decomposed to meet the energy metabolism demand, rather than fat, when the duration of low temperature is extended^22^. Specially, glycerol and urea, which are important intermediates of fat and protein metabolism, respectively, indirectly reflect the metabolism of fat and protein in fish at low temperatures. Besides being the substrate for liver gluconeogenesis, glycerol can be used as an energy substrate to produce ATP and provide energy through oxidative phosphorylation in mitochondria^23^. Further, amino acids are the main material for glycerol synthesis at low temperatures, in which case, ammonia produced by amino acids is converted into urea. It has been reported that an increase in urea concentration in blood effectively brings toxins out of the fish at low temperature, maintaining the homeostasis of cells^7^. In the present study, it was found that the urea content in the plasma of *L*. *crocea* first decreased and then increased, whereas plasma-glycerol content decreased gradually, suggesting that protein might gradually replace fat and became the energy source for metabolism in *L*. *crocea* under an extended duration of low temperature stress. Further, fish respiration rate and oxygen consumption decreased with decreasing water temperature, which resulted in an overall decrease in metabolic rate^3^. Similarly, here, we found that the contents of glycerol and urea in the plasma decreased greatly upon acute low temperature stress, compared with those of the control group, indicating that the rates of fat and protein metabolism in *L*. *crocea* were slowed down at cold temperatures, providing a guarantee for long-term survival in case of a lack of food resources in low temperature environments.

TG, the main form of fat storage, is hydrolyzed to produce FFA and glycerol at low temperatures to meet the metabolic demand^1^. It has been reported that the concentration of plasma TG in fish first increased and then decreased, following exposure to low temperature stress^24,25^. Similarly, here we found that TG concentration in the plasma of *L*. *crocea* increased significantly at 0 h of acute low temperature stress, and then decreased rapidly. Furthermore, many studies have found that the greater the lipid storage in fish, the higher its overwintering survival rate^26–28^. Therefore, the increase of plasma TG in the study suggested that the fat was mobilized and catabolized to provide energy, thus coping with the sudden drop in temperature. However, TG content in the plasma decreased gradually under an extended duration of low temperature stress, which might be due to the fact that liver damage inhibited the synthesis of TG in the liver.

During the process of fat metabolism, glycerol is transported through the intestinal epithelium and then enters the liver through blood circulation, participating in liver metabolism^29^. It has been reported that AQP7 and AQP10 play an important role in the absorption and release of glycerol in the liver and intestine, and regulate the production of blood glucose^30,31^. Furthermore, the concentration of plasma glycerol in AQP7 knockout mutant mice was relatively low, compared to that of normal mice^32,33^. Here, we found that the expression level of the *aqp7* gene in the heart and liver, as well as the expression level of the *aqp10* gene in the intestine, both decreased under acute low temperature stress, suggesting that the absorption and release of glycerol in the heart, liver and intestines was limited, thereby decreasing the rate of glycerol metabolism. AQPs reportedly promote uptake of glycerol into muscle fibers in humans, which is then converted into glycerol-3-phosphoric acid via phosphorylation by creatine kinase, and is eventually converted into glycogen or lactic acid as energy for muscle contraction^34^. Furthermore, AQP7 is a functional glycerol channel protein that plays a key role in the myocardial function in mice^35^. The levels of glycerol and ATP in the heart of *aqp7* knockout mutant mice decreased, which led to the decrease of glycerol metabolism and the production of energy^35^. It has been reported that energy metabolism and contractility in ectothermic vertebrate hearts slow at low temperatures^36^. Thus, the decrease in expression level of the *aqp7* gene inhibits glycerol metabolism, which may be one of the reasons of the slow metabolism of the heart at low temperatures, but due to lack of reliable evidence, further study is needed. The continuous decrease in expression level of the *aqp7* and *aqp10* genes, as well as the concentration of glycerol in the plasma led to a reduction in glycerol metabolism with the extension of low temperature stress, which might inhibit TG synthesis and gluconeogenesis in the liver.

The liver is the largest high metabolic-rate organ in living organisms and plays important roles in digestion, excretion, detoxification, and immunity; the integrity of the hepatocyte membrane is important for these physiological functions of the liver. However, hepatocyte membranes are susceptible to adverse environmental factors^37,38^. It has been reported that the hepatocyte density in *Cyprinus carpio* var. *songpu mirror* decreased after overwintering, with the atrophy and rupture of hepatocytes^39^. It has also been found that the hepatocyte density in nile tilapia (*Oreochromis niloticus*) liver decreases obviously, and the whole cell shape is not seen, as well as being accompanied by the vacuolization of cells under cold environments^40^. Similarly, here we found that the hepatocytes in *L*. *crocea* became large, and the cell density decreased, with vacuolization and rupture of some hepatocytes after 48 h of acute low temperature stress. These observations suggested that hepatocyte necrosis in *L*. *crocea* caused liver damage, thereby hindering normal liver metabolism and reabsorption of glycerol and TG through blood circulation. Further, the rupture of the hepatocyte membrane might induce a decrease in the expression level of the *aqp7* gene under acute low temperature stress, thereby inhibiting glycerol absorption and release. This might be a potential reason resulting in a continuous decrease of plasma glycerol under low temperature.

In the process of glycerol metabolism, the intestine plays an important role in the reabsorption of glycerol. Additionally, the intestine plays key roles in water and electrolyte balance, endocrine regulation of digestion and metabolism, and immunity^41^. The intestinal mucosa, used as a barrier, prevents various pathogens and harmful substances from entering the intestinal tissue^42–44^. The free surfaces of the columnar epithelial cells (absorptive cells, ACs) in the intestinal epithelium have a clearly striated border composed of microvilli. The outer membrane of the microvilli is covered with a thick cell coat, mainly composed of glycoproteins produced by the ACs, which bind to the proteins and lipids of the plasma membrane to protect cells. In addition, the folds of intestinal GCs are scattered among ACs and can secrete mucoproteins to protect the intestinal mucosa from injury^42,43^. However, the permeability of the intestinal mucosa increases and harmful substances and bacteria enter the intestinal tissue more easily under stress, resulting in damage to the barrier in the intestinal mucosa^45^. Here, we showed that the breaks in the intestinal folds caused incompletion or shedding in the striated border, with a decrease in the number of GCs and vacuolization of partial lamina propria at 48 h under acute low temperature stress. Similarly, Yu *et al*.^39^ found that the number of GCs in Songpu mirror carp obviously decreased, and the striated border began to experience atrophy during overwintering. It was evident that the integrity of the intestinal mucosa was damaged under continuous low temperature, resulting in increased permeability, which then led to various pathogens and harmful substances more easily entering into the intestinal tissue, and dysfunction of the intestinal digestive system, barrier, and immunity, causing inflammation and damage to the intestine in *L*. *crocea*. At the same time, a continuous decrease in *aqp10* expression in the intestine also reflected a decrease in glycerol absorption rate in the intestine.

However, fish can normally move and feed after overwintering, suggesting that damage to high metabolic-rate tissues caused by low temperature is not irreversible. Interestingly, the integrity of the barrier of the intestinal mucosa is rapidly reconstructed upon transfer to a suitable environment, even if it has been severely damaged^42^. Moreover, mucoproteins secreted by GCs also play a role in epithelial repair and mucosal healing^46^. Similar to the intestine, the liver also shows a high regenerative capacity^47^. Here, we found that the number of GCs on the intestinal mucosa increased while vacuolization of the lamina propria and width of the submucosa increased after 48 h of rewarming. Meanwhile, the hepatocyte density increased, and the volume of hepatocytes was reduced, together with a relative decrease in vacuolated and broken cells, compared to the situation in the liver under low temperature. Additionally, we found that the concentrations of glycerol, BUN, and TG in plasma had basically returned to the control levels at 12 h of rewarming. In addition, the expression levels of *aqp7* and *aqp10* genes in the heart, liver, and intestine respectively, basically returned to those of the control group within 24 h of rewarming, suggesting that the normal metabolism of fat and protein in *L*. *crocea* were rapidly activated after rewarming, thereby ensuring normal feeding and metabolism. This is in close agreement with reports of a rapid increase in fish metabolic rates with increasing water temperature^48^. From the above results, it was suggested that *L*. *crocea* has a remarkable self-repair ability, thereby ensuring normal digestion and metabolism after overwintering.

## Conclusions

In the present study, we explored the effects of acute low temperature stress on the metabolism of *L*. *crocea* as reflected by plasma biochemical indices and morphological and gene expression (*aqp7* and *aqp10*) changes. Further, its recovery ability of post-cold stress was also studied when *L*. *crocea* returned to a suitable temperature for growing. We found that *L*. *crocea* quickly adapted to low temperature environments by reducing fat and protein metabolism, which was one reason explaining the fish survive for months, in the absence of natural food and diminished feeding capacity over winter. Furthermore, the decrease in the expression of *aqp7* and *aqp10* genes might reduce the energy metabolism of *L*. *crocea* under low temperature, sustaining a low metabolic level; this suggested that *aqp7* and *aqp10* genes might be used as candidate genes responsible for the adaptation of fish to low temperature. However, the damage to the liver and intestines under continuous low temperature stress suggested that the adaptation of *L*. *crocea* to low temperature was limited. Interestingly, it showed remarkable post-cold stress recovery at a suitable temperature for growing, ensuring that its metabolism was restored to normal levels, providing a guarantee for the normal activity and feeding of *L*. *crocea* after overwintering.

## Material and Methods

### Ethics statement

This study did not involve human or non-human primate subjects. The research was carried out in strict accordance with the requirements of the “Governing Regulation for the Use of Experimental Animals in Zhejiang Province” (Zhejiang Provincial Government Order No. 263, released in August 17, 2009, effective from October 1, 2010). The Institutional Animal Care and Use Committee at the Zhejiang Laboratory Animal Research Center and Ningbo University approved the study.

### Animals and experimental design

In all, 270 *L*. *crocea* individuals with an average body weight of 60 ± 5 g were obtained in late May 2016 from the Science and Technology Innovation Base of Ningbo Ocean and Fisheries, Zhejiang Province, China, and kept in a round pool (5,000 L) in sea water at 23 °C for a 15-day adaptation period before the start of the experiment. Fish were fed a specific mix food for *L*. *crocea* (Tianma Science and Technology Group Co., Ltd., Fujian, China), twice a day, 50 g each time during this period. After the adaptation period, fish were placed in six plastic cylinder-type, temperature-controlled, circulating water tanks (300 L) with sea water at 23 ± 0.5 °C, with 45 fish in each tank. The six water tanks were divided into two groups of three tanks each (control and low temperature). Water temperature in the low temperature tanks was decreased at 1 °C·h^−1^ from 23 °C to 9 °C. When the temperature reached 9 °C, the time was recorded as 0 h. Blood samples were taken from the fish through the caudal vein using a heparinized syringe and were placed in 1.5 mL RNase-free microcentrifuge tubes at 0, 6, 12, 24, and 48 h after low temperature stress treatment initiation. Tissue samples from the brain, gill, heart, kidney, liver, spleen, intestine, gonad, and muscle, were collected from the fish anesthetized by tricaine methanesulfonate (MS-222) (Kangyihe Raw-food Material Co., Ltd., Wuhan, China). Finally, samples were immediately frozen in liquid nitrogen and stored at −80 °C until use. Six fish were sampled from each group at each sampling time-point (i.e., two fish were randomly sampled from each treatment tank).

After 48 h under low temperature stress, water temperature of the low temperature group was returned to 23 °C within 30 min and then maintained at 23 ± 0.5 °C. The time was recorded as 0 h of the rewarming treatment group when the temperature reached 23 °C. As described above, tissues and blood were sampled at 6, 12, 24 and 48 h from both groups. In order to minimize the influence of factors other than temperature, no food was given for the duration of the experiment.

### Determination of plasma glycerol, BUN, and TG

Blood samples were centrifuged at 4 °C, at 7,500 × g for 15 min to separate the plasma, which was stored below −20 °C until use. Plasma urea and glycerol were measured using the BUN and glycerol assay kits (Jiancheng Bioengineering Institute, Nanjing, China), respectively. Plasma triglyceride was determined using a triglyceride assay kit (Jiancheng Bioengineering Institute, Nanjing, China) following the instructions of the manufacturer. Three biological replicates were measured for each group.

### Sample collection for paraffin sectioning and hematoxylin and eosin (HE) staining

Intestines and livers from fish in the control and treatment groups were dissected after the experiment, cut into cubes, and fixed in Bouin’s fixative (containing 75 mL of saturated picric acid buffer solution (25 mL of 40% formaldehyde, and 5 mL of 100% acetic acid) over less than one month. Thereafter, the samples were rinsed in 70% ethanol and stored until further processing. Samples were cut into slices for dehydration, clearing, encapsulation, and HE staining, and were observed under a light microscope (Olympus BX51, Japan). Images were acquired using Image-Pro Plus 6.0 image analysis software. Six biological replicates were processed from each group.

### Total RNA extraction and quantitative real-time polymerase chain reaction (qPCR) analysis

Total RNA was extracted from flash-frozen tissues with TRIzol reagent (Tiangen, Beijing, China) following the instructions of the manufacturer. Total RNA was reverse-transcribed using the Prime Script™ RT reagent kit (TaKaRa, Dalian, China).

Quantitative real-time PCR was performed for expression analysis of *aqp7* and *aqp10* genes in control tissues (brain, gill, heart, kidney, liver, spleen, intestine, gonad, and muscle) of *L*. *crocea*, as well as expression level of *aqp7* (XM_010733506) in the heart and liver along with *aqp10* (XM_019264297) in the intestine, over stress duration. Transcript of the β-actin (ADN52693) gene was used as the internal control. Primers for qRT-PCR are listed in Table 2. Real-time quantitative PCR was run using a SYBR green Master I (Roche, Basel, Switzerland) on a Roche LightCycler 480 (Bioplastics, Holland); PCR was performed as follows: 94 °C for 5 min; 40 cycles at 94 °C for 20 s, 60 °C for 20 s, 72 °C for 20 s. Real-time quantitative PCR reaction was performed in triplicate for each sample and the mean value was used to calculate mRNA levels. Three biological replicates were measured for each group. Transcript levels of the relative quantities of *aqp7* and *aqp10* genes were ascertained as follows: ΔCt treatment (threshold cycle of *aqp7* and *aqp10* of treated fish) = Ct treatment − Ct β-actin treatment; ΔCt control (threshold cycle of *aqp7* and *aqp10* of control fish) = Ct control − Ct β-actin control; ΔΔCt = ΔCt treatment − ΔCt control; and the relative quantity levels of *aqp7* and *aqp10* mRNA expressions were calculated using the 2^−ΔΔCt^ method^49^.

**Table 2:** Primers used for expression analysis of *aqp7* and *aqp10* genes in *L*. *crocea*.

Primer name	Nucleotide sequence (5′ → 3′)
*aqp7*-F	TTTGTTCGTGTGGGACTTGC
*aqp7*-R	AACACCCAGTCCAAAACCCA
*aqp10*-F	ACCACGACTCAAGATAAGAAGGG
*aqp10*-R	GCCCAGGAAGCATAAACTCAG
β-Actin-F	TTATGAAGGCTATGCCCTGCC
β-Actin-R	TGAAGGAGTAGCCACGCTCTGT

### Statistical analysis

Data were analyzed using SPSS 17.0 and Microsoft Excel software. All data are expressed as mean ± SD. Independent-sample t-test was performed to determine any significant differences between the treatment and control groups. *P* values < 0.05 were considered statistically significant. All plots were made in OriginPro 8.5 (OriginLab, Northampton, MA).
